# Experiments on Muriatic Acid Gas, with Observations on Its Chemical Constitution, and on Some Other Subjects of Chemical Theory

**Published:** 1818-06

**Authors:** John Murray

**Affiliations:** Fellow of the Royal College of Physicians of Edinburgh


*Experiments on Muriatic Acid Gas, with Observations on its Chemical Constitution, and on some other Subjects of Chemical Theory.* By John Murray, M.D. F.R.S.E. Fellow of the Royal College of Physicians of Edinburgh. Quarto, pp. 42. Edinburgh, 1818.*

We hold it an auspicious circumstance that we have it in our power, ere we commence our trimestral labours, to lay before our readers an outline of this paper, the production of one of the most distinguished chemists of the day;—a paper which, without hesitation, we pronounce to be one of the ablest that has appeared for a series of years, and destined, probably, to effect as great a revolution in chemical theory as the brilliant discoveries of Lavoisier, or the scarcely less happy researches of Davy. In bestowing so large a share of notice, as we mean to do, on

* From the Transactions of the Royal Society of Edinburgh, read the 15th of December 1817, and 5th of January 1818.

this admirable Essay, let it not be said, that we depart from the wholesome plan of restriction on which we have pledged ourselves, that this Journal shall be rigorously conducted. No doubt we have promised to be "chiefly practical," and it is a promise which we shall unquestionably abide by: all speculation—however ingenious—when it is merely speculation, we do and shall most steadily reject, as being useless in itself and unprofitable to our readers. But when, as on the present occasion, important doctrinal points and fundamental principles of chemical science are discussed, all classes in the profession, we apprehend, from the youngest to the most advanced, from the lowest to the highest, from "the hewers of wood and drawers of water" to the more favoured few who have arrived at "the golden age" of professional success, and who rest the "honours of their head" on the fulness of their fame—all, we say, must take an interest in the matter, and must lend us their ready attention, convinced that we have not abused their confidence, or trifled with their time. The collection, too, which contains this Essay, can come in the way of but a very small portion of our readers; and this forms an additional reason, if reasons were wanting, for our devoting to it such a number of our pages.

Without farther preface, we proceed to our proper business.—The paper before us is divided into two parts: The 1st. containing a detail of farther experiments on the nature of oxymuriatic and muriatic acids, in prosecution of the controversy about their constitution, which Dr. Murray has, for some years past, sustained against Sir Humphrey Davy. The 2d. contains general reasonings and conclusions, in which the author most ingeniously proves that, throughout the whole compass of chemistry, hydrogen is an acidifying principle equal to oxygen in power, and in generality of operation.

Of the first part we shall say comparatively little; because we feel, that betwixt such combatants as Sir Humphrey Davy and Dr. Murray, it would be rash and highly presumptuous in us to interpose our critical authority. Indeed, we conceive the matter quite beyond the scope of Medical Journalists, unless they be more than ordinarily imbued in chemical philosophy. For our parts, we are content to look on in submissive and unaffected neutrality, with subdued feelings of respect and wonder—feelings somewhat analogous to those with which the imagination is filled on reading the contests of superior natures, so powerfully depicted by the daring genius of Milton.

All our readers must be aware, that Sir Humphrey Davy considers oxymuriatic acid a simple substance, which, from its greenish colour, he calls chlorine; and that its union with hydrogen forms, as the sole product, muriatic acid gas: the latter, by consequence, must be a compound of chlorine and hydrogen. Dr. Murray, on the other hand, is a strenuous supporter of the old doctrine, which holds, that oxymuriatic acid is a compound of muriatic acid with oxygen; and that muriatic acid, in its gaseous state, contains combined water, which is essential to its constitution, and amounts to one-fourth of its weight. As decisive of the rival merits of these opposite opinions, Dr. Murray proposed the experiment of procuring water from muriate of ammonia, formed by the combination of dry ammoniacal and muriatic acid gases: because, since ammonia contains no oxygen, if water is obtained from its combination with muriatic acid gas, the result cannot be accounted for on Sir H. Davy's hypothesis, but must be regarded as a proof of the presence of water in the acid gas; and as a proof equally conclusive of the existence of oxygen in oxymuriatic acid.

This experiment was accordingly performed by Dr. M. by combining thirty cubic inches of muriatic acid gas, with the same volume of ammoniacal gas, carefully dried. The salt formed was exposed in a small retort, with a receiver adapted to it, to a moderate heat gradually raised. Moisture speedily condensed in the neck of the retort, which increased and condensed into small globules.

"This result (says Dr. M.) was admitted by those who defend the new doctrine, water being obtained, it was allowed, "in no inconsiderable quantity." But to obviate the conclusion, it was asserted that this is water which has been absorbed by the salt from the atmosphere, or that it might be derived from hygrometric vapour in the gases submitted to experiment. This was affirmed by Sir. H. Davy, but the reverse I was able to demonstrate by farther experimental investigations." P. 3.

After proving that the salt absorbs no moisture from the air in its ordinary state of dryness, and after adverting to the fact established by the experiments of Henry and Gay Lussac, that muriatic acid gas contains no hygrometric vapour, and that no quantity of the latter that can be assumed, would be adequate to account for the quantity of water actually obtained, Dr. Murray instituted another set of experiments, which he notices thus:—" Another form of experiment occurred to me, still more direct and simple, viz. that of transmitting muriatic acid, in its gaseous form, over ignited metals. If water be obtained in this experiment, it is a result which would prove subversive of the new doctrine; for muriatic acid gas is held to be the real acid, free from water; and the only change which can happen, is that of the metal decomposing the acid, attracting its chlorine, and liberating its hydrogen. And the experiment is farther free from the only resource which remained to the advocates of that doctrine, in the case of water being obtained from muriate of ammonia, that it might be derived from the decomposition of the elements of ammonia, regarding it as an alkali containing oxygen. If water were really obtained from the combination of muriatic acid and ammoniacal gases, it would rather indicate, it was said, the decomposition of nitrogen, than the existence of water as a constituent of muriatic acid. No weight, I believe, is due to such an assumption; but if any importance were attached to it, it is precluded, if water is obtained from the action of metals on muriatic acid gas.

" I have executed the experiment in several forms; and in all with a more or less satisfactory result." P. 10.

The experiments were made with the filings of iron, and of zinc: the muriatic acid gas employed was always extricated from materials carefully dried; the gas itself was farther dried by transmission over an extensive surface of loose muriate of lime, and at last collected over dry mercury. In short, every step of the process seems to have been devised by our author with the utmost ingenuity, and yet with the utmost fairness: and in every experiment, he contends, that water has been procured from the muriatic acid gas. He adds, " it is obvious that such a result cannot be accounted for on the hypothesis that it is the real acid free from water, a compound merely of chlorine and hydrogen. On the opposite doctrine, as muriatic acid in its gaseous form is held to contain water, it may be supposed to afford a portion of it." P. 15.

The celebrated author next inquires, whence the water so obtained is derived; and it is an inquiry involved in much greater difficulty than might, at first sight, be expected. On his own doctrine, it is evident that no water could, *a priori*, be expected; because, in the action of metals on the muriatic acid gas, the combined or constitutional water of the latter is decomposed, its oxygen going to oxidate the metal (which then combines with the real acid), and its hydrogen being evolved. This is what we would be prepared to expect—and nothing but this. The difficulty is equal on Sir H. Davy's hypothesis: muriatic acid gas is composed of chlorine or oxymuriatic gas and hydrogen. Now, a metal acting on it must attract the oxymuriatic acid and liberate the hydrogen. No water, therefore, ought to appear more on this theory than the other; but the real products in both must be a dry muriate, or chloride and hydrogen gas. Here, then, the question still recurs, how the production of the water is to be accounted for?

Did our limits permit, we would right gladly follow the able author into that train of profound and highly ingenious argumentation by which he solves this very serious difficulty. He proves, first, that the water is not the result of hygrometric vapour; and, secondly, that it does not proceed from extrinsic water existing (not constitutionally, but still, as might be supposed, chemically combined) in the muriatic acid gas, seeing many facts prove that this gas can receive no additional portion of water beyond that which is essential to its constitution. He makes it appear, thirdly, that the difficulty cannot be got over by supposing that the metal may require less oxygen than is supposed, in combining with the acid, and that a portion of water will thus remain undecomposed, to be deposited; because the latter supposition is contrary to the law which regulates the combination of metallic oxides with acids—namely, that the quantity of acid entering into union is proportional to the quantity of oxygen, so that if an oxide were formed, in any case, at a lower degree of oxidation, it would only combine with a proportionably smaller quantity of acid, and the quantity of combined water detached from the combination would be the same.

The explanation of the difficulty which the author gives is a most ingenious, and, so far as we can judge, a most acute one; it is this—that the metallic oxide attracts more *real* acid, so as thus to liberate a larger proportion of water. We shall subjoin his own words, which make his meaning quite clear.

"These results appear to establish the production of a *super*-muriate in the action of these metals on the acid, and this accounts for the appearance of a portion of water, since, supposing water to exist in muriatic acid gas, the quantity combined with that proportion of acid which would establish a neutral compound, is the quantity required to oxidate the metal to form that compound; and if any additional portion of acid enter into union, (as always takes place in these cases) the water of this must be liberated, or be at least capable of being expelled by heat." P. 21.

Dr. M. reminds his readers, that whether the explanation proposed is satisfactory or not, still the *fact* of the production of water in these cases is not invalidated by the theoretical difficulty; and that on the old hypothesis, this fact, even were we unable to explain the *modus operandi,* would be only a difficulty; whereas, on Sir H. Davy's hypothesis, it is an impossible result.

With a calm remark, such as the above, the author takes leave of the controversy.—He conducts himself throughout with an air of quiet dignity highly deserving of imitation in all matters of science which happen to become the subject of controversy.

We now proceed to Part Second, wherein the author developes his profound and most ingenious views with regard to the extensive agency of hydrogen as a principle of acidity in bodies. Here, however, our analysis shall chiefly consist of extracts.

" When water is obtained from muriatic acid gas, it does not necessarily follow, that it has pre-existed in the state of water. It is equally possible, *a priori,* that its elements may be present in simultaneous combination with the acid, or its radical—that the acid is a ternary compound of a radical with oxygen and hydrogen; and that it is decomposed in those processes by which water is procured, the hydrogen with the requisite proportion of oxygen, combining to form water; and its radical, with an excess of oxygen, remaining in union with the substance by which the change has been effected." P. 25.

" Muriatic acid gas, then, according to this doctrine, is the real acid, a ternary compound of a radical (at present unknown) with oxygen and hydrogen, exactly as sulphuric acid, in its highest state of concentration, is the real acid, a ternary compound of sulphur, oxygen, and hydrogen. When it is submitted to an alkaline base, the action exerted causes its decomposition; its hydrogen and part of its oxygen combine to form water, and its radical, with its remaining oxygen, unite with the base, forming a neutral compound, analogous to what other acids of similar constitution form. When a similar result is obtained from the action of a metal, its whole oxygen must be considered as retained, and its hydrogen is liberated.

" Nitric acid, in its highest state of concentration, is not a definite compound of real acid, with about a fourth of its weight of water; but a ternary compound of nitrogen, oxygen, and hydrogen. Phosphoric acid is a triple compound of phosphorus, oxygen, and hydrogen; and phosphorous acid is the proper binary compound of phosphorus and oxygen. The oxalic, tartaric, and other vegetable acids, are admitted to be ternary compounds of carbon, oxygen, and hydrogen, and are therefore in strict conformity to the doctrine now illustrated. P. 26.

It was Lavoisier who first inferred, by ample induction, that oxygen is a principle of acidity: and it was Berthollet who first suggested that hydrogen also is occasionally so, being led to this conclusion by the acid nature of sulphuretted hydrogen, and the Prussic acid. The very modern discovery by Gay Lussac, of the radical cyanagen (which is convertible into Prussic acid by the addition of hydrogen) and of iodine and its relations with hydrogen, have still farther sanctioned these views. But it is to Dr. Murray that the praise is due, of having first discovered hydrogen as an acidifying principle in circumstances, situations, and combinations, where it was never suspected heretofore: and of having explained the new and important axiom, that whilst acidity is sometimes exclusively connected with oxygen, sometimes with hydrogen—it is more frequently the result of their combined operation.

" There appears sufficient reason to infer, that from the united action of these elements, a higher degree of acidity is acquired than from the action of either alone. Sulphur affords a striking example of this. With hydrogen, it forms a weak acid (sulphuretted hydrogen; with oxygen, it also forms an acid (the sulphurous); which, though of superior energy, still does not display much power. With hydrogen and oxygen, it seems to receive the acidifying influence of both, and its acidity is proportionally exalted.

" Nitrogen with hydrogen forms a compound altogether destitute of acidity, and possessed even of qualities the reverse. With oxygen, in two definite proportions, it forms oxides; and it is doubtful if, in any proportion, it can establish, with oxygen, an insulated acid. But, with oxygen and hydrogen in union, it forms nitric acid, a compound more permanent, and of energetic action.

" Carbon, with hydrogen, forms compounds which retain inflammability without any acid quality: with oxygen, it forms first an inflammable oxyde, and with a larger proportion a weak acid. But, combined with both hydrogen and oxygen, in different proportions, it forms, in the vegetable acids, compounds having high acidity. These acids, therefore, are not to be regarded, according to the theory of Lavoisier, as composed of a compound base of carbon and hydrogen, acidified by oxygen, but of a simple base, carbon, acidified by the joint action of oxygen and hydrogen." P. 27, 28.

The learned author goes on, with wonderful acuteness and clearness of reasoning, to apply the fundamental views thus broached by him, to all the acids and their bases, to the muriatic and the oxymuriatic acids, and to the compounds formed by the latter with sulphur and phosphorus, —to iodine in its relations with oxygen and hydrogen, and to the explanation of some facts, whose solution on the received hypothesis is either faulty or fallacious. Our limits will only permit us to hint at the various matters discussed; yet, we cannot help extracting the following general conclusions:

" The view which I have now illustrated, is not to be regarded as mere speculation. The evidence in support of it is just as conclusive as that from which the opposite opinion is inferred. The obtaining water from a compound is no necessary proof that water pre-existed in it; and conversely, the causing water to enter into combination in a compound, is no necessary proof that it remains in the state of water in the product. In many cases, we draw the reverse conclusions, considering water as being formed where it is obtained, and as decomposed where it is communicated. And in the case of its relation to acids, it will be found, that there is no strict evidence of its existing as water in combination with what is considered as the real acid; and of course the conclusion is equally open to be drawn, that it exists in these combinations in the state of its elements, and that when obtained, it is a product of a change of composition."

" It is even more probable, *a priori*, that the ultimate elements should act on each other where energetic affinities are evidently exerted, than the immediate principles; and the relations of these elements will determine the combinations and the proportions. And by admitting this view, we avoid the anomaly which is presented in ascribing to the agency of water effects so different from those to which it usually gives rise. In general, water dilutes or dissolves bodies, or absorbs gases, without otherwise modifying their properties, or communicating to them any important chemical powers: but, in the particular cases now referred to, it is supposed to produce the effects of the most energetic chemical agent. Certainly, it is much more probable that such effects should arise from the action of elements so powerful as oxygen and hydrogen, than from the agency of water, which, in its general relations, exerts such feeble powers." P. 35, 36.

From all this, it follows, that the principle on which the presence of combined water in acids has been supposed to depend, viz. the strong attraction of the acid to water, is altogether fallacious; for on this principle, sulphurous acid should contain combined water, and sulphuretted hydrogen, and even carbonic acid, might be expected also to retain some, but they do not. The fact, therefore, evidently depends on difference of constitution. Upon the whole, we think that Dr. M. has proved by the strongest probable evidence—and let it be remembered that *probable* evidence, and not that of demonstration, is the sort on which the principles of chemistry rest—that in the constitution of acids there is a simultaneous combination of the *elements* of the water and of the acid, and not a combination of water and acid.

The author next ingeniously contends, that the same general views may be extended to the alkalis as well as the acids. Here, again, his own words will best explain his reasonings:

"Alkalinity, as well as acidity, is the result, apparently, of the action of oxygen; the fixed alkalis, the earths, and the metallic oxides, which all contain it as a common element, forming a series in which it is difficult to draw any well-defined line of distinction. Ammonia alone remains an exception: it contains no oxygen, and yet possesses in a very marked degree all the alkaline properties,—an anomaly so great as to have led every chemist to infer that oxygen must exist as an element in one or other of its constituent principles; and as nitrogen is the one apparently least elementary, it has been supposed to be a compound containing oxygen. The result may be accounted for, however, on a very different principle. As hydrogen gives rise, in some cases, as well as oxygen does, to *acidity*, so it may, in other cases, give rise to *alkalinity*. Under this point of view, ammonia is a compound of which nitrogen is the base, deriving its alkaline power from hydrogen; it stands, therefore, in the same relation to the other alkalis, that sulphuretted hydrogen does to the acids. And thus the whole speculation, with regard to the imaginary metallic base *ammonium,* and the existence of oxygen in ammonia and in nitrogen, falls to the ground ; while the anomaly presented by the alkali is removed. If the claim of the lately discovered principle in opium, *morphia* as it has been named, to the distinction of an alkali be established, as from its origin it must probably have a compound base, it may, if it contain hydrogen, bear the same relation to the other alkalis that Prussic acid does to the acids; or if it contain oxygen, it will be analogous to the vegetable acids." P. 39.

Here, too, as in the case of the acids, Dr. M. contends, that combined water, or rather the combination of the principles of water, in alkalis, depends on relations subsisting among the ultimate elements, not on an affinity exerted by the alkali itself; and hence the conclusion, that these elements are in ternary union is additionally strengthened.—Speaking of the alkalis and alkaline earths, he says,

" Their superior alkaline energy, compared with the common metallic oxides, may obviously arise from the joint action of the hydrogen and oxygen, in the same manner that the acidity of the ternary, compared with the binary acids, is increased by a similar constitution. Thus the class of alkalis will exhibit the same relations as the class of acids." P. 40.

He next makes some ingenious observations as to the neutral salts, upon this new view; but our limits will not permit us to condense them.

We here close our outline of this highly interesting paper: the extracts we have given will sufficiently vindicate the assertion we hazarded at the outset of the article, that it is one of the ablest papers that has appeared for a series of years. Notwithstanding the unusual scope we have taken, we have done it great injustice ; nay, we fear that it may appear to our readers aphoristical and gratuitous in its conclusions, simply because we have been obliged, generally, to state *merely* the conclusions, suppressing, for the most part, the beautiful inductive reasoning by which these conclusions are warranted. But this was hardly to be avoided, since every line is surcharged with ideas, and every sentence deep and full of matter. *This* being the case, we would earnestly recommend the perusal of the Essay itself.

